# Abnormal white‐matter rich‐club organization in obsessive–compulsive disorder

**DOI:** 10.1002/hbm.25984

**Published:** 2022-06-23

**Authors:** Samantha Baldi, Stijn Michielse, Chris Vriend, Martijn P. van den Heuvel, Odile A. van den Heuvel, Koen R. J. Schruers, Liesbet Goossens

**Affiliations:** ^1^ Department of Psychiatry and Neuropsychology School for Mental Health and Neuroscience, Maastricht University Maastricht Netherlands; ^2^ Department of Neurosurgery School for Mental Health and Neuroscience, Maastricht University Maastricht Netherlands; ^3^ Department of Anatomy and Neurosciences Amsterdam UMC, Vrije Universiteit Amsterdam, Amsterdam Neuroscience Amsterdam Netherlands; ^4^ Department of Psychiatry Amsterdam UMC, Vrije Universiteit Amsterdam, Amsterdam Neuroscience Amsterdam Netherlands; ^5^ Department of Complex Trait Genetics Center for Neurogenomics and Cognitive Research, Vrije Universiteit Amsterdam Amsterdam Netherlands

**Keywords:** connectivity, diffusion‐weighted imaging, obsessive–compulsive disorder, probabilistic tractography, rich‐club organization, structural networks

## Abstract

Rich‐club organization is key to efficient global neuronal signaling and integration of information. Alterations interfere with higher‐order cognitive processes, and are common to several psychiatric and neurological conditions. A few studies examining the structural connectome in obsessive–compulsive disorder (OCD) suggest lower efficiency of information transfer across the brain. However, it remains unclear whether this is due to alterations in rich‐club organization. In the current study, the structural connectome of 28 unmedicated OCD patients, 8 of their unaffected siblings and 28 healthy controls was reconstructed by means of diffusion‐weighted imaging and probabilistic tractography. Topological and weighted measures of rich‐club organization and connectivity were computed, alongside global and nodal measures of network integration and segregation. The relationship between clinical scores and network properties was explored. Compared to healthy controls, OCD patients displayed significantly lower topological and weighted rich‐club organization, allocating a smaller fraction of all connection weights to the rich‐club core. Global clustering coefficient, local efficiency, and clustering of nonrich club nodes were significantly higher in OCD patients. Significant three‐group differences emerged, with siblings displaying highest and lowest values in different measures. No significant correlation with any clinical score was found. Our results suggest weaker structural connectivity between rich‐club nodes in OCD patients, possibly resulting in lower network integration in favor of higher network segregation. We highlight the need of looking at network‐based alterations in brain organization and function when investigating the neurobiological basis of this disorder, and stimulate further research into potential familial protective factors against the development of OCD.

## Graphical abstract

The present study investigates rich‐club organization and rich‐club connectivity as potential markers of OCD, by using probabilistic tractography to reconstruct the white‐matter network of a group of unmedicated OCD patients. Our results suggest weaker structural connectivity between rich‐club nodes in OCD patients, possibly resulting in lower network integration in favor of higher network segregation.

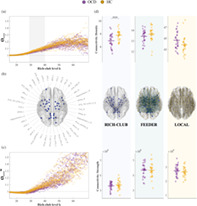

## 1 INTRODUCTION

Obsessive–compulsive disorder (OCD) is a severe psychiatric condition affecting 2%–3% of the population world‐wide, characterized by the combined or isolated presence of intrusive, recurrent thoughts, and associated repeated behaviors or mental rituals (Association & Association, 2013). Despite the abundance of evidence that has accumulated in the last decades, the exact neurobiology of OCD remains elusive; neuroanatomical models attempting to identify dysfunction of one or a few isolated brain regions are labelled as too simplistic, and it has long been recognized that looking at networks is the key (Shephard et al., 2021). Graph‐theoretical analyses of magnetic resonance imaging (MRI) data have proven valuable to understanding how information is integrated and communicated throughout the brain as a complex network, and have been widely employed to study several neurological and psychiatric conditions (Cao et al., 2015; Griffa et al., 2013).

In the normal functioning brain, spatially and functionally distinct regions exchange information quickly and efficiently, facilitating cognitive and behavioral responses appropriate to the environmental demands. Key players in this process are network hubs, regions that display high connectivity to the entire network, but are first and foremost densely interconnected with each other. This ensemble of connections forms a “rich‐club” within the brain, a high‐capacity structural core that allows information to travel across distant regions that would hardly communicate otherwise (van den Heuvel et al., 2012). Rich‐club connections have been mainly characterized by macroscopic white‐matter connections (van den Heuvel et al., 2012; van den Heuvel & Sporns, 2011), although studies have also linked structural and functional rich‐club organization with each other (Grayson et al., 2014; Senden et al., 2014; van den Heuvel & Sporns, 2013). Regions belonging to the rich‐club have been shown to span all major resting‐state networks (RSNs), and to participate in a large proportion of inter‐RSNs connections (van den Heuvel & Sporns, 2013). For this reason, the rich‐club is regarded as the anatomical substrate for efficient communication across distant and/or segregated functional systems (van den Heuvel & Sporns, 2013), argued to significantly contribute to global neural integration (Senden et al., 2014; van den Heuvel et al., 2012; Vértes et al., 2014) and healthy brain function (Baggio et al., 2015; Ball et al., 2014). Given its prominent role, alterations in rich‐club organization are believed to interfere with higher‐order cognitive processes, leading to behavioral dysfunction. Such alterations are reported for several neurological and psychiatric conditions, including Alzheimer's disease (Dai et al., 2019), Parkinson's disease (Hall et al., 2018), schizophrenia (van den Heuvel et al., 2013), major depression disorder (Wang et al., 2019), autism spectrum disorder and attention‐deficit/hyperactivity disorder (Ray et al., 2014).

A few studies examining white‐matter networks in OCD suggest altered efficiency of information transfer across the brain (Peng et al., 2021; Reess et al., 2016; Zhong et al., 2014; Zhou et al., 2021). One study reports lower global and regional efficiency in OCD patients, predominantly within fronto‐striatal and fronto‐parietal networks (Zhong et al., 2014). Another study points to a cluster of lower connectivity comprising temporo‐limbic, insular, orbitofrontal and striatal regions. Their analysis of graph measures highlights local alterations of mainly temporo‐limbic regions, with indications of lower efficient connectivity of the left amygdala in particular (Reess et al., 2016). Remarkably, neither of these studies have investigated whether the reported decrease in efficiency of information transfer could relate to alterations in the rich‐club organization of the brain. An attempt in this direction has been made recently by Zhou et al. (2021), who show in contrast with findings above, higher global efficiency and higher rich‐club organization and rich‐club connectivity in OCD patients. The authors suggest that long‐distance information integration and transmission capacity might be enhanced, potentially as a result of compensatory mechanisms (Zhou et al., 2021). These results have however not been replicated by Peng et al. (2021), who report lower rich‐club organization and rich‐club connectivity in OCD patients. Their findings of similar alterations in a group of unaffected first‐degree relatives support altered rich‐club organization as a candidate vulnerability marker of OCD (Peng et al., 2021).

The thorny problem of connectome‐based studies is the myriad of methodological choices that stand between the construction of the network and the implementation and interpretation of graph measures. Contradicting findings are often blamed on the technical diversity of the study that generated them, rarely questioning the true biological validity of what is being explored. However, when there is no gold standard set out to follow, and each technical choice has its own pro and counterarguments, the scientific reliability of any result lies within their stability and replicability across a variety of methodological nuances. The limited and contradicting findings available to date do not suffice for a clear understanding of the rich‐club phenomenon in this patient population, but more research is clearly needed.

The present study adds to the discussion and investigates rich‐club organization and rich‐club connectivity as potential markers of OCD, by using probabilistic tractography to reconstruct the white‐matter network of a group of unmedicated OCD patients. Further, we included the preliminary analyses of a small sample of unaffected first‐degree relatives, with the aim of prompting further research into familial vulnerability. We hypothesized that OCD patients and their unaffected siblings would show abnormal rich‐club organization and rich‐club connectivity in their white‐matter network.

## 2 METHODS AND MATERIALS

### 2.1 Participants

The study included 44 patients diagnosed with OCD who were medication‐free for at least 4 weeks at the time of enrolment (mean age 38.5 ± 9.9 year), 15 of their unaffected siblings (SIB, mean age 38.1 ± 14.1) and 37 healthy controls (HC, mean age 39.5 ± 11.5 year), matched on age, sex and education level. Details about the sample and recruitment have been described elsewhere (Fan et al., 2016). Briefly, patients were excluded in case of current psychoactive medication use, current or past psychosis, current or past alcohol usage disorder, major physical or neurological illness or in the presence of MRI contra‐indications. Psychiatric comorbidity did not constitute a reason for exclusion, as long as the primary diagnosis was OCD, without predominant hoarding. Clinical characteristics were assessed with the Yale‐Brown Obsessive Compulsive Scale (Y‐BOCS, symptom list and severity scale) (Goodman et al., 1989), the Obsessive–Compulsive Inventory‐Revised (OCI‐R) (Foa et al., 2002) and the Montgomery‐Åsberg Depression Rating Scale (MADRS) (Montgomery & Åsberg, 1979). All participants were screened on axis I psychiatric disorders using the Structural Clinical Interview for DSM‐IV‐TR Axis I Disorders (First, 2014). Siblings should not meet lifetime criteria for OCD nor for any psychiatric diagnosis. Healthy controls had no current psychiatric diagnosis nor a family history of OCD.

### 2.2 MRI acquisition

MRI was performed using a 3‐Tesla MR system (Signa HDxt, GE Healthcare, Milwaukee, USA) equipped with an eight‐channel phased‐array head coil. Diffusion‐weighted echo‐planar imaging (Lidstone et al., 2010) was collected at 30 randomly distributed diffusion weighted (*b* = 1000 s/mm^2^) and five reference (*b* = 0 s/mm^2^) volumes with 49 axial slices at 2.4 mm thickness covering the whole brain (repetition time TR = 14,000 ms, echo time TE = 85 ms). The acquired in‐plane resolution was 2.0 × 2.0 mm^2^, which was reconstructed to 1.0 × 1.0 mm^2^. Parallel imaging was applied with an acceleration factor of 2. Structural images were acquired using a 3D sagittal T1‐weighted sequence (TR = 7.8 ms, TE = 3 ms, TI = 450 ms, FlipAngle = 12, voxel size 1.0 × .0.977 × 0.977 mm^3^, 172 slices).

### 2.3 Image preprocessing

Diffusion MRI data were preprocessed using the FMRIB Software Library (FSL version 6.0; http://www.fmrib.ox.ac.uk/fsl) and Advanced Normalization Tools (ANTs version 3.0; http://stnava.github.io/ANTs/). Images were corrected for motion and eddy current‐induced susceptibility distortions by applying affine alignment of each diffusion‐weighted image to the mean *b* = 0 image (Andersson & Sotiropoulos, 2016). EPI‐induced distortions correction was performed by nonlinear registration of the DWI to T1 (Wang et al., 2017), using ANTs' symmetric normalization *SyN* registration algorithm (Avants et al., 2008). We visually inspected the output of registration for all participants.

FSL's *bedpostX* (Behrens et al., 2007) was used to estimate the voxel‐wise diffusion parameter distributions. We ran *probtrackx2* for probabilistic fiber tracking with crossing fibers with the following parameters: 2000 steps per samples with a steplength of 0.5 mm, curvature threshold of 0.2 and volume fraction set to 0.1, sampling a total of 5000 streamline fibers per voxel and keeping all other default parameters. We corrected path distribution for the length of the pathways.

Tracking was performed by seeding from 210 bilateral cortical regions and 36 bilateral subcortical regions obtained from the Brainnetome Atlas (Fan et al., 2016). Adding to this set, the subthalamic nucleus and the bed nucleus of the stria terminalis were obtained from the Subthalamic Nucleus Atlas (Forstmann et al., 2012) and from a probabilistic map of the National Institute of Mental Health (https://afni.nimh.nih.gov), respectively. All seeds (*n* = 250) were registered from standard to native space following previously described methods (Gong et al., 2009). Briefly, T1 images were registered to the MNI1521 mm brain template using ANTs' *SyN* registration tools (Avants et al., 2008). *antsApplyTransforms* was used to warp the cortical and subcortical seeds from MNI to native space by concatenating the inverse of warp fields and generic affine matrix using *GenericLabel[Linear]* as interpolation method. All masks were thresholded (at 50) and binarized.

### 2.4 Network construction

For each participant, a brain network was reconstructed with the cortical and subcortical seeds representing its *nodes*, and the white‐matter tracts interconnecting them representing its *edges*. For each pair of nodes, the value of each edge was assigned as the number of reconstructed streamlines (NOS). First, the arithmetic average of NOS connecting node (i, j) and that connecting (j, i) was obtained to create an undirected network. Next, proportional thresholding of the network edges was applied by retaining only a proportion (.23) of the strongest network edges (Tijms et al., 2012). Further, only those edges that were present in at least 60% of all group members were retained, calculated per group separately (de Reus & van den Heuvel, 2013). Next to NOS‐weighted networks, binary networks were computed (i.e., thresholded edge weights were set to 1, 0 otherwise). The stability of the results was checked using proportional thresholds of .30 and .55 (Supplementary Material).

### 2.5 Network characteristics

All graph measures were computed using the Brain Connectivity Toolbox (Rubinov & Sporns, 2010) in Matlab (Matlab R2019b; Mathworks Inc). Basic network characteristics such as network density (i.e., fraction of present connections to possible connections, ignoring edge weights) and overall network connectivity (i.e., sum of edge weights across all nodes) were compared between groups for the raw and thresholded networks. Following the two‐step thresholding procedure, obtained networks were checked to preserve key properties of biological networks, namely connectedness (i.e., >80% of nodes being connected to at least another node) and small‐world topology (i.e., a small‐world index >1) (Lynall et al., 2010). Global and local graph measures of efficiency and clustering coefficient were computed on the weighted networks (Supplementary Material).

### 2.6 Rich‐club analysis

**FIGURE 1:**
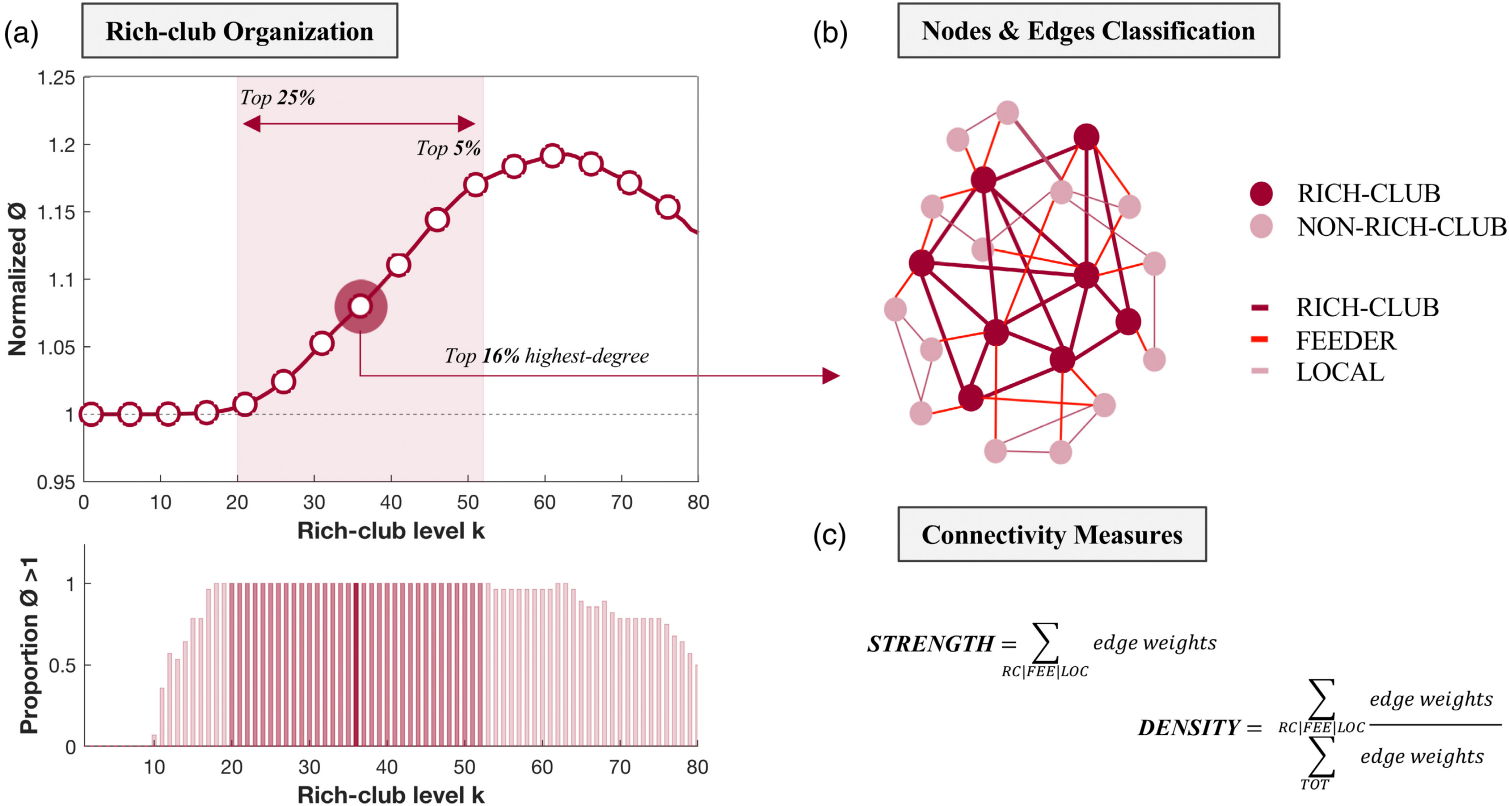
Schematic representation of the rich‐club analysis. First, rich‐club coefficients Ø (unweighted and weighted) are calculated at increasing rich‐club levels *k* and normalized by the averaged rich‐club curve of a set of comparable random networks. A schematic representation of the normalized groups average rich‐club curve is shown (*a top*). Normalized Ø > 1 (dashed line in *a top*) indicates significant rich‐club organization in a network. The bar graph represents the proportion p of participants for which this holds true across rich‐club levels (*p* = 1 indicates that all participants display significant rich‐club organization) (*a bottom*). Next, the nodes of the network are classified into rich‐club or nonrich‐club nodes (*b*). Members of the rich‐club are defined as the most highly connected nodes of the network common to all participants. Main results are reported for the top 16% highest‐degree regions, but a wider range is considered, including from the top 25% to the top 5% highest‐degree regions (red shaded area in *a top and bottom*). Network edges are classified accordingly into rich‐club (connections between rich‐club nodes), feeder (connections between rich‐club and nonrich‐club nodes) and local (connections between nonrich‐club nodes) (*b*). Two connectivity measures are finally computed; connectivity strength represents the sum of all edge weights within each connection class, and weighted connectivity density represents the ratio of the connectivity strength of each connection class to the connectivity strength of the whole brain (*c*). FEE, feeder connections; LOC, local connections; RC, rich‐club connections; TOT, whole‐brain connections

A schematic representation of the rich‐club analysis steps is shown in Figure 1.

#### 2.6.1 Rich‐club organization

Computing the rich‐club coefficient Ø at degree level of *k* allows characterizing the rich‐club behavior of a network (van den Heuvel & Sporns, 2011). In the topological, unweighted rich‐club (Ø), rich‐club nodes preferentially create connections between each other. In the weighted rich‐club (Ø^w^), rich‐club nodes preferentially allocate the strongest weights to the connections between them (Alstott et al., 2014). The empirical Ø and Ø^w^ were computed on the binary and NOS‐weighted networks respectively, and normalized by the averaged rich‐club curve of a set of comparable random networks (Ø_rand_) (van den Heuvel & Sporns, 2011). For each network, a population of 1000 random networks was generated by rewiring the edges of the original matrix, while preserving its connection density, degree and strength distribution (Rubinov & Sporns, 2011). A normalized coefficient Ø_norm_ >1 (calculated as Ø/Ø_rand_) is indicative of rich‐club organization in a network (Colizza et al., 2006) and is assigned a (one‐sided) p‐value by calculating the proportion of Ø_rand_ that exceeded the empirically measured metric Ø (FDR‐corrected at q = 0.05).

#### 2.6.2 Nodes classes: rich‐club vs non‐rich club

Rich‐club nodes (i.e., nodes with degree > *k*) were defined for each participant as the top 16% (*k* > 38) highest‐degree regions (van den Heuvel & Sporns, 2011). The regions classified as rich‐club nodes common to all participants were then selected as the final set and used for subsequent analyses, as previously reported (van den Heuvel & Sporns, 2011). This was done separately for the two‐group (OCD vs. HC, *n* = 56) and the three‐group (*n* = 64) comparisons. Analyses were repeated considering smaller and larger sets of hub regions (including from the top 5% to the top 25% highest degree‐regions) (Supplementary Material).

#### 2.6.3 Connections classes: rich‐club, feeder and local

Structural connections between nodes were classified accordingly into rich club (i.e., between rich‐club nodes), feeder (i.e., between rich‐club and non‐rich‐club nodes) and local (i.e., between non‐rich‐club nodes) connections. Two measures of connectivity were calculated for each connection class and compared between groups. Connectivity *strength* was defined as the sum of all edge weights (i.e., sum of all NOS) within each connection class. Weighted connectivity *density* was defined as the ratio of the connectivity strength of each connection class to the connectivity strength of the whole brain, representing an index of network topology (van den Heuvel et al., 2012).

### 2.7 Statistical analysis

ANOVA was used to compare age, years of education, sex, and clinical variables between groups. Comparisons of network characteristics were performed using nonparametric permutation testing for randomizing group assignment (separately for OCD‐HC, OCD‐SIB, SIB‐HC) (Krol, 2020). The 50,000 permutations of group assignments yielded an empirical null distribution of effects under the hypothesis of no difference between groups. The measured difference was assigned a (two‐sided) p‐value, as the percentage of the computed null distribution greater than or equal to the empirically measured metric. The same procedure was followed to compare rich‐club measures between OCD patients and controls. We separately tested for ordered differences between the three groups using the Jonckheere‐Terpstra test (Supplementary Material). Group comparisons of rich‐club coefficients were iterated over the range of increasing k displaying significant rich‐club organization (Ø_norm_ >1) for at least 97% of participants, and a false‐discovery rate (FDR) threshold of q = .05 was applied on the obtained p‐values. Spearman's partial correlation coefficients were calculated in the OCD group to investigate the relationship between rich‐club measures and clinical variables (Y‐BOCS and OCI‐R total and subscores, disease duration and MADRS), while controlling for age, sex and education. The area under the curve (AUC) was computed for rich‐club, feeder and local measures across the range of increasing *k* considered for the between‐group comparisons.

## 3 RESULTS

### 3.1 Demographic and clinical characteristics

Following visual inspection of the raw images, 16 OCD patients, 7 unaffected siblings and 9 healthy controls were excluded from subsequent analyses, due to positioning of the field of view resulting in (major) cuts of the bilateral temporal pole. The final sample thus included a total of 28 OCD patients (mean age 36.8 ± 9.2 year), 8 unaffected siblings (mean age 37.8 ± 13.2 year) and 28 healthy controls (mean age 40.6 ± 11.0 year). Age, sex and education did not differ significantly between the three groups (Table 1). OCD patients displayed significantly higher Y‐BOCS, OCI‐R, and MADRS scores compared to their unaffected siblings and controls (Table 1).

**TABLE 1:** Demographic and clinical characteristics of OCD patients, their unaffected siblings and healthy controls

	OCD patients (*n* = 28)	Siblings (*n* = 8)	HC (*n* = 28)	Analysis
	Mean (±SD)	Mean (±SD)	Mean (±SD)	*F* (*p* value)
*Demographic variables*				
Age^1^	36.8 (±9.2)	37.8 (±13.2)	40.6 (±11.0)	0.99 (.378)
Education^1^	13.6 (±3.4)	12.3 (±2.4)	12.2 (±2.8)	1.55 (.219)
Sex (M/F)	11/17	4/4	12/18	0.14 (.866)
				
*Clinical variables*				
Disease duration^1^	22.7 (±11.3)	0	0	66.5 (<.001)
Y‐BOCS (total score)	21.3 (±6.0)	0.1 (±0.4)	0	220 (<.001)
Y‐BOCS obsessions	10.3 (±3.6)	0.1 (±0.4)	0	146 (<.001)
Y‐BOCS compulsions	11.0 (±3.0)	0	0	236 (<.001)
OCI‐R (total score)	22.8 (±11.7)	2.9 (±2.9)	3.3 (±5.4)	37.3 (<.001)
OCI‐R washing	3.0 (±3.6)	0	0.4 (±0.7)	7.97 (<.001)
OCI‐R checking	6.4 (±4.2)	0.6 (±0.9)	0.3 (±0.6)	32.5 (<.001)
OCI‐R ordering	4.4 (±3.7)	0.4 (±0.7)	0.9 (±1.8)	12.4 (<.001)
OCI‐R obsession	4.9 (±3.5)	0.6 (±1.2)	0.4 (±1.6)	21.1 (<.001)
OCI‐R hoarding	1.8 (±2.6)	1.1 (±1.4)	1.2 (±2.2)	0.4 (0.67)
OCI‐R neutralizing	2.1 (±3.0)	0.1 (±0.4)	0.1 (±0.2)	7.46 (<.001)
MADRS	9.3 (±6.9)	1.3 (±2.9)	1.0 (±1.6)	20.6 (<.001)

*Note*: 1 expressed in years.

Abbreviations: HC, healthy controls; MADRS, Montgomery‐Åsberg Depression Rating Scale; OCI‐R, obsessive–compulsive inventory revised; SD, standard deviation of the mean; Y‐BOCS, yale‐brown obsessive–compulsive scale.

### 3.2 Network characteristics

No significant differences in network density (*p* = .26) and overall network connectivity (*p* = .53) emerged, both looking at the raw and thresholded networks of OCD patients and healthy controls (Table S1). Compared to the latter, OCD patients displayed significantly higher global clustering coefficient (*p* = .03), but no differences in global efficiency (*p* = .64) or small world‐topology (*p* = .82) (Table S1). OCD patients also displayed significantly higher local efficiency for *n* = 7 nodes and higher local clustering for *n* = 74 nodes (*q* < .05), spanning extensive frontal and temporal areas, cingulate cortex, lateral occipital cortex and subdivisions of the thalamus, among others (Table S2). Unaffected siblings presented with significantly lower overall network connectivity (*p* < .001), lower global efficiency (*p* < .001) and lower global clustering coefficient (*p* < .001) compared to OCD patients and healthy controls. On the other hand, following the two‐step network thresholding procedure, siblings displayed significantly higher network density (*p* < .001). The details and implications of these results are outlined in the Supplementary Material.

### 3.3 Rich‐club organization

**FIGURE 2:**
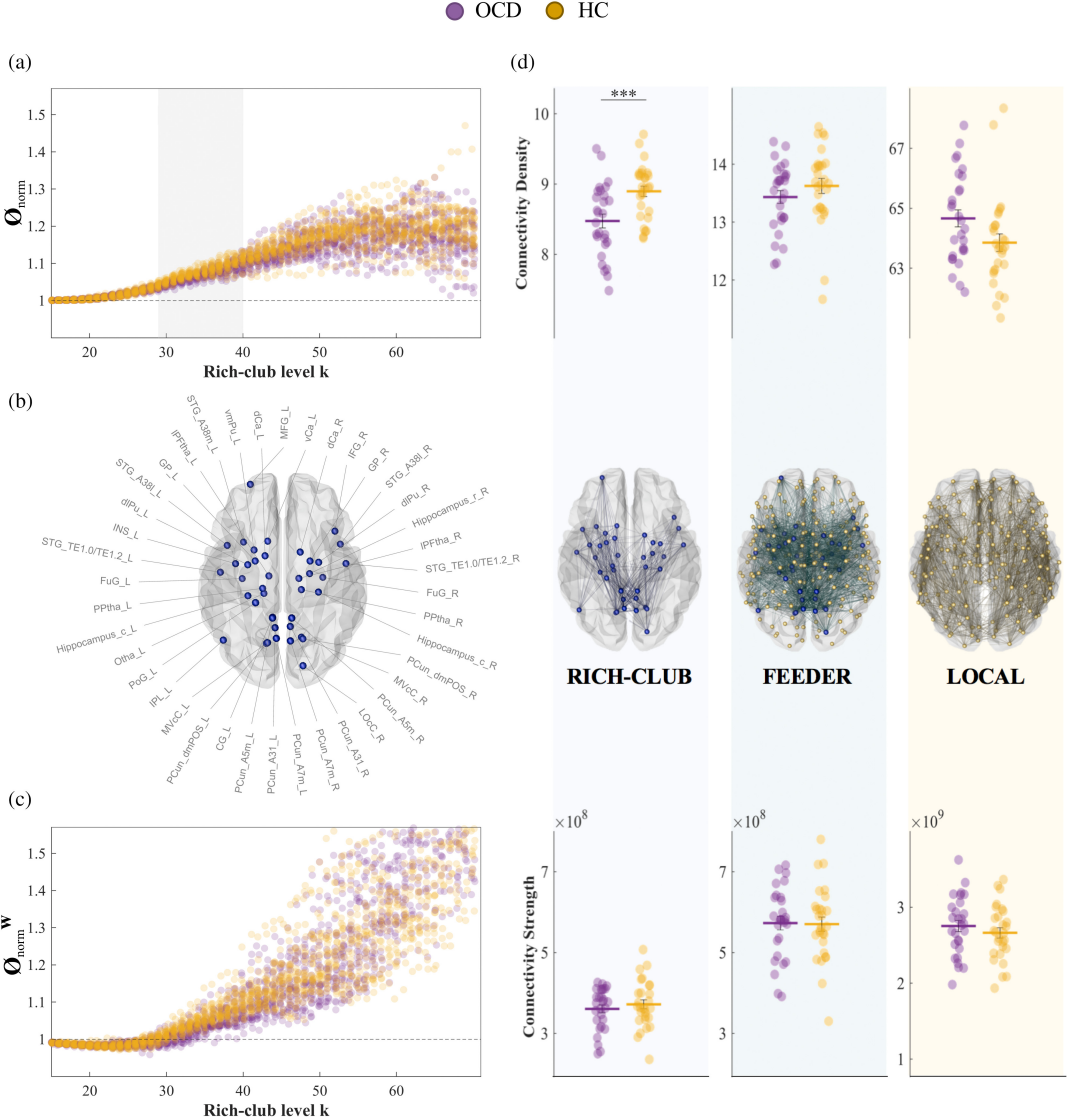
Individual normalized Ø and Ø^w^ are plotted for OCD patients (purple) and healthy controls (yellow) for different rich‐club levels. Normalized Ø > 1 (dashed line in *a* and *c*) indicates significant rich‐club organization. The grey shaded area indicates where rich‐club organization of the two groups is significantly different (*p* < .01, FDR‐corrected) (*a*, *c*). Rich‐club nodes are selected as the top 16% highest‐degree nodes of the network (*b*), and network edges are classified accordingly into rich‐club, feeder and local (*d middle*). Topological (i.e., connectivity density; *D top*) and weighted (i.e., connectivity strength; *D bottom*) properties are calculated for each connection class and compared between groups. ***p < .001. CG, cingulate gyrus; dCa, dorsal caudate; dlPu, dorsolateral putamen; FuG, fusiform gyrus; GP, globus pallidus; HC, healthy controls; IFG, inferior frontal gyrus; INS, insular gyrus; IPL, inferior parietal lobule; L, left; LOcC, lateral occipital cortex; lPFtha, lateral prefrontal thalamus; MFG, middle frontal gyrus; MVcC, medioventral occipital cortex; OCD, obsessive–compulsive disorder patients; Otha, occipital thalamus; PCun, precuneus; PoG, postcentral gyrus; PPtha, posterior parietal thalamus; R, right; STG, superior temporal gyrus; vCA, ventral caudate; vmPu, ventromedial putamen

Rich‐club organization was found in the white‐matter networks of both OCD patients and healthy controls (Ø_norm_ range *k* = 20–53; Ø_norm_^w^ range *k* = 42–63) (Figure 2a,c). Compared to controls, OCD patients displayed significantly lower topological rich‐club organization (range *k* = 29–40, *q* < .01, Hedge's *g* = [.81, 1.48]). OCD patients also displayed lower weighted rich‐club organization for the range k = 42–56, although this difference was not significant (*q* > .34). A significantly lower weighted Ø_norm_^w^ was found when using proportional thresholds of .30 and .55 (Figure S1).

The three‐group analysis revealed significant ordered differences between groups for both topological (HC > OCD > SIB, range *k* = 20–53, *q* < .05) and weighted (SIB > HC > OCD, *k* = 42–58, *q* < .05) rich‐club organization (Supplementary Material, Figure S3).

### 3.4 Connectivity strength and density of rich‐club, feeder and local connections

Consistent with previous reports, rich‐club nodes selected at the top 16% highest‐degree nodes across OCD patients and controls included the middle and inferior frontal gyrus, superior temporal gyrus, fusiform gyrus, inferior parietal lobule, precuneus, postcentral gyrus, insula, cingulate gyrus, ventral and lateral occipital cortex and, subcortically, the hippocampus, (posterior parietal, occipital and lateral prefrontal) thalamus and regions of the basal ganglia (caudate, putamen, and globus pallidus) (Peng et al., 2021; van den Heuvel & Sporns, 2011; Zhou et al., 2021) (Figure 2b).

No significant differences between groups were found in the connectivity *strength* of either rich‐club (*p* = .45), feeder (*p* = .91) or local (*p* = .37) connections (Figure 2d bottom). OCD patients displayed significantly lower rich‐club weighted connectivity *density* (*p* < .001, Hedge's *g* = .94) and a trend to increased local connectivity density (*p* = .052, Hedge's *g* = .53) compared to controls. No significant differences were observed for feeder connectivity density (Figure 2d top). Results were stable when using proportional thresholds of .30 and .55 (Figure S2).

Unaffected siblings displayed the highest rich‐club and local connectivity density, but the lowest feeder connectivity density (Supplementary Material, Figure S3).

### 3.5 Correlations with clinical characteristics

The AUC computed for rich‐club organization (Mean [±SD]: Ø_norm_ = 35.71 [±0.37], Ø_norm_^w^ = 26.05 [±3.25]), and for strength and weighted density of rich‐club ([Mean [±SD]: strength = 4.65e + 09 [±6.84e + 08], density = 109.13 [±6.98]), feeder ([Mean [±SD]: strength = 9.05e + 09 [±1.37e + 09], density = 212.23 [±9.16]) and local ([Mean [±SD]: strength = 5.82e + 10 [±8.2e + 09], density = 1.37e + 03 [±23.67]) connections was correlated with clinical characteristics (Y‐BOCS and OCI‐R total and subscores, disease duration and MADRS, Table 1), while controlling for age, sex and education. No significant correlations are reported (Table S3).

## 4 DISCUSSION

The current study used probabilistic tractography to investigate white‐matter rich‐club organization in OCD. Compared to healthy controls, OCD patients displayed significantly lower unweighted and, to some extent, weighted rich‐club organization, suggesting that brain network hubs exhibit less connections between them, and do not necessarily allocate the strongest weights thereto (Alstott et al., 2014). OCD patients congruously displayed significantly lower rich‐club weighted connectivity *density*, representing a smaller fraction of all connection weights allocated to the rich‐club compared to the healthy counterpart. On the other hand, no differences emerged between groups when comparing connectivity *strength* in absolute terms, neither for whole‐brain, rich‐club, feeder nor local connections. Thus, our results mostly point to differences in the topological arrangement of connections and their weights to the rich‐club, rather than absolute differences in the strength of such connections. While still being able to draw on comparable resources, OCD patients might manage their connectivity system differently, allocating more weight to peripheral connections at the detriment of a central core of hub nodes. Consistent with this hypothesis, OCD patients also displayed a trend to increased local weighted connectivity density, meaning that a higher fraction of all connection weights is allocated to local connections compared to healthy controls.

Rich‐club organization is regarded as a marker of network integration, allowing distant regions to quickly and effectively exchange information between each other (van den Heuvel & Sporns, 2013). Efficient brain networks however stem from the delicate balance between integration and segregation of functions, thus equally relying on the specialization of regions, or clusters of regions, to take on specific tasks (Rubinov & Sporns, 2010). In patients with OCD, the scale might be tipped in favor of a more segregated network. As opposed to lower rich‐club organization, we found OCD patients to display significantly higher global clustering coefficient, local efficiency and local clustering specifically of non‐rich‐club nodes. These results are consistent with previous evidence of decreased rich‐club connectivity (Peng et al., 2021) and decreased global efficiency as opposed to increased clustering (Peng et al., 2021; Zhong et al., 2014), while disagreeing with the pattern described by Zhou et al. (2021), pointing to higher measures of network efficiency in OCD. After all, the susceptibility of graph measures to specific methodological choices cannot be neglected. The methods used by the present and previous studies differed considerably on multiple fronts. As opposed to deterministic tractography, we employed probabilistic tractography to map the connectomes of OCD patients. Furthermore, we used a brain parcellation with a higher resolution compared to what previously used, a methodological difference of the known potential impact on connectivity measures (Carmi et al., 2019; Messé, 2020). Nonetheless, despite the technical differences, results across studies point to altered efficiency of information transfer across the brain, yet awaiting for further research to clarify the nature of rich‐club organization anomalies in OCD.

For both OCD patients and healthy controls, rich‐club nodes included areas of frontal, parietal, temporal and occipital cortices, next to subdivisions of the insula and cingulate cortex, and subcortical regions of the basal ganglia, thalamus and hippocampus, to a large extent consistent with what previously reported (Peng et al., 2021; van den Heuvel & Sporns, 2013; Zhou et al., 2021). The literature linking dysfunctional nodes of the cortico‐striatal‐thalamo‐cortical loops to OC symptomatology is extensive and includes several lines of evidence, ranging from early positron‐emission tomography studies demonstrating metabolic abnormalities to volumetric, functional and lesion studies, all pointing to the critical involvement of the frontal as well as subcortical components of these circuits (Milad & Rauch, 2012). Beyond this traditional view, a potentially central role has been ascribed to the dorsal anterior cingulate cortex (dACC), hub in the cognitive control and fear learning and extinction networks, exerting control signals via extensive connections with surrounding cortical and subcortical structures to direct behavioral responses (McGovern & Sheth, 2017). Not only central to a mechanistic theory on the emergence of obsessions and compulsions, the dACC is also the target of surgical and neuromodulatory treatment interventions (Fineberg et al., 2020; McGovern & Sheth, 2017), placing it under the spotlight of OCD brain dynamics. Additionally, many of the identified rich‐club nodes critically participate in RSNs like the default mode, salience and frontoparietal networks, the inter‐ and intra‐connectivity of which has consistently been reported altered in OCD (Gürsel et al., 2018). However, despite the overlap between rich‐club nodes and the regions generally implicated in OCD pathophysiology, the question arises about the specificity of rich‐club organization anomalies to OCD. No significant correlation between any rich‐club measure and OCD‐specific clinical characteristics were found in neither the present nor some of the previous studies (Peng et al., 2021; Reess et al., 2016). A recent meta‐analysis comprising almost 900 patients across 12 neurological and psychiatric disorders found that rich‐club connections were disproportionally affected across disorders compared to peripheral connections (de Lange et al., 2019), and independent studies reporting altered rich‐club organization in single disorders are numerous (Dai et al., 2019; Hall et al., 2018; Ray et al., 2014; van den Heuvel et al., 2013; Wang et al., 2019). It has been suggested that, because of their central embedding in the network, central regions and connections might be not only particularly vulnerable to various disease processes themselves (Buckner et al., 2009), but also at increased risk of propagating these processes across the network (Fornito et al., 2015; Iturria‐Medina et al., 2014). Given the importance of central connections for appropriate cognitive function (Cees De Groot et al., 2000; van den Heuvel & Sporns, 2011), any defect that might affect this system could then result in various forms of cognitive impairment. Considering that deficits in cognitive function overlap across disorders (Millan et al., 2012), it is possible that abnormal rich‐club organization constitutes a transdiagnostic vulnerability marker to psychopathology in general, mediating dysfunctional traits common across diagnostic categories rather than specific symptoms. Future studies should further address this hypothesis, trying to identify unique and/or common cognitive markers relating to rich‐club dysfunction in OCD with respect to other brain disorders.

Alternatively, the absence of disease severity effects could point to rich‐club organization anomalies being trait rather than state markers of OCD. Family‐based studies are valuable tools to unravel putative vulnerability markers of a disorder, indexing a genetic liability and allowing the dissection of state and trait signatures. The present study preliminarily investigated rich‐club organization in a small group of unaffected siblings. Generally, neuroimaging markers of anomaly present in both patients and unaffected first‐degree relatives, but not in healthy controls, are good candidates, and potential endophenotypes have been identified in measures of white‐matter microstructure (Dikmeer et al., 2021; Fan et al., 2016; Menzies et al., 2008), network properties (Peng et al., 2014) and functional patterns of dysconnectivity (Chamberlain et al., 2008; Peng et al., 2014; Vaghi et al., 2017). Research on whether rich‐club organization could be considered as such is limited, with only one study reporting intermediate levels of rich‐club connectivity in unaffected siblings (Peng et al., 2021). Our results however revealed a pattern of higher weighted rich‐club organization and rich‐club density in unaffected siblings compared to OCD patients and healthy controls, as opposed to lower unweighted rich‐club organization. Although limited in their generalizability by the small sample size, our results point to a buffering mechanism that unaffected first‐degree relatives may put in place. Namely, they might recruit additional resources (in terms of a higher fraction of all connection weights) to preserve cognitive performance and mental health in spite of a reduction in the number of rich‐club connections (i.e., unweighted rich‐club organization). Although no previous research investigated changes in the trade‐off between topological and weighted rich‐club measures, the idea of a buffering mechanism is congruous with e.g. reports of increased resting‐state connectivity between cognitive control networks in unaffected siblings (de Vries et al., 2019). However, longitudinal and developmental studies are needed to correctly place rich‐club organization along the trajectory to OCD manifestation or, if considered as transdiagnostic marker, psychopathology in general.

The current study has some limitations. First, due to fitting of the field of view during MRI acquisition resulting in major cuts of the temporal pole, many participants were excluded from the current analyses, reducing the available sample size. Despite the strong effect sizes reported, future studies should aim to include larger samples. Specially, findings concerning the group of unaffected siblings are to be taken with extreme caution, and are mostly intended to suggest hypotheses that could be addressed by future studies. Additionally, inherent limitations of the DWI sequence and probabilistic tractography urge us to interpret the results carefully. More advanced protocols such as multi‐shell procedures will allow the implementation of processing and tractography methods offering better control over the biological plausibility of the reconstructed white‐matter pathways (Smith et al., 2012).

## 5 CONCLUSIONS

We investigated rich‐club organization in a sample of unmedicated OCD patients. Our results suggest a topological shift of connections and their weights away from the rich‐club, resulting in weaker structural connectivity between network hubs. Preliminary findings of increased rich‐club organization in unaffected siblings hint at a neuroimaging feature worth investigating further in the context of familial vulnerability or resilience to developing the disorder. We finally underscore the importance of looking at network‐based alterations in brain organization and function when investigating OCD.

## CONFLICT OF INTEREST

Odile A. van den Heuvel received a one‐time consultation fee (2021) from Lundbeck. The other authors declare that they have no conflict of interest.

## Supporting information


**Appendix S1** Supporting information

## Data Availability

The data and analysis code in this manuscript are available from the corresponding author upon reasonable request.
