# Supplementary material for: Abnormal white‐matter rich‐club organization in obsessive–compulsive disorder
Source: Hum Brain Mapp. 2022 Jun 23;43(15):4699–709. doi: 10.1002/hbm.25984 (PMC9491289; doi:10.1002/hbm.25984)
Supplement: Supplementary file 1 — Appendix S1 Supporting information [file HBM-43-4699-s001.docx]

Supplementary Material of

**Abnormal White-matter Rich-club Organization in Obsessive-compulsive Disorder**

Samantha Baldi, Stijn Michielse, Chris Vriend, Martijn P. van den Heuvel, Odile A. van den Heuvel, Koen R.J. Schruers, Liesbet Goossens

**Network characteristics**

Network density (i.e. fraction of present connections to possible connections, ignoring edge weights) and overall network connectivity (i.e. sum of edge weights across all nodes) were first computed for the raw networks (i.e. before any thresholding procedure was applied). Permutation testing (50,000 permutations) revealed no significant differences between OCD patients and healthy controls in neither of these measures (see **Table S1**). Following the two-step thresholding procedure, the networks of both groups of subjects showed to preserve connectedness (> 80% of nodes connected to at least one other node) and small-world topology (small-world index > 1). Again, no significant differences between groups were detected for neither network density, overall network connectivity or small-worldness (see **Table S1**).

Global efficiency (i.e. the average of inverse shortest path length) and global clustering coefficient (i.e. average clustering coefficient across all nodes of the network) were additionally computed on the thresholded weighted networks, employing the “efficiency_wei.m” and “clustering_coef_wu.m” functions from the Brain Connectivity Toolbox, respectively. No differences were detected between the global efficiency of OCD patients and healthy controls, whereas OCD patients displayed significantly increased global clustering coefficient (p = .03, see **Table S1**).

Local efficiency and local clustering coefficients were calculated for each node of the weighted network (n = 250). P-values obtained with permutation testing were corrected for multiple comparisons (FDR at q = .05). OCD patients displayed significantly increased local efficiency in the left orbital and postcentral gyrus, and in the right superior temporal gyrus, superior parietal lobule and lateral occipital cortex. The right middle and inferior temporal gyrus, and the right posterior superior temporal sulcus displayed significantly reduced local efficiency in OCD patients (see **Table S2**). Notably, all these nodes belonged to the pool of non-rich-club nodes. A total of n = 74 nodes displayed significantly increased clustering coefficient in OCD patients compared to healthy controls, and are listed in Table S2. The majority of these nodes (n = 58) again belonged to non-rich-club nodes. The right middle and inferior temporal gyrus, and the right posterior superior temporal sulcus displayed significantly decreased clustering coefficient in OCD patients (see **Table S2**).

**Validation analyses: smaller and larger sets of rich-club nodes**

For the two-group comparison (OCD vs. HC), rich-club nodes were selected as the nodes with a degree k > 38, including the top 16% highest-degree nodes of the network common to all patients and controls (n = 56). However, smaller and larger sets of rich-club nodes were also considered, including nodes with a degree k > 32 (top 25%) to k > 51 (top 5%). Rich-club, feeder and local connectivity *strength* and weighted *density* were calculated across this range, and are displayed in **Figure S2**.

Results were consistent across k levels; no significant differences emerged for neither rich-club, feeder nor local connectivity *strength*. OCD patients displayed significantly reduced rich-club weighted connectivity *density* for the range k > 32 (top 25%) to k > 42 (top 11%) (p < .05, Hedge’s g = [0.64, 0.94]). Nevertheless, the observed difference between all measures followed a similar trend across rich-club sets, regardless of statistical significance.

**Validation analyses: proportional thresholding parameters**

The stability of the results was checked using proportional thresholds of .30 and .55 to construct NOS-weighted and binary networks.

***Threshold .30***. Rich-club organization was found in the white-matter network of both OCD patients and healthy controls (Ø_norm_, range k = 27 to k = 87; Ø_norm_^w^, range k = 47 to k = 140). OCD patients displayed significantly reduced topological rich-club organization (range k = 35 to k = 58, q < .05, Hedge’s g = [0.72,1.71]; range k = 65 to k = 70; q < .05; Hedge’s g = [0.61,0.94]; k = 75, q = .049; Hedge’s g = [0.59]; k = 77, q = .04; Hedge’s g = [0.63]; k = 80, q = .02; Hedge’s g = [0.74]; k = 83, q = .04; Hedge’s g = [0.64]). OCD patients also displayed significantly reduced weighted rich-club organization (range k = 47 to k = 55, q < .05, Hedge’s g = [0.54,0.79]) (**Figure S1**).

Rich-club nodes selected at the top 16% (k > 53) highest-degree nodes at the group level included the same brain regions reported in the main text. Rich-club, feeder and local connectivity strength and connectivity density were computed across a range of rich-club levels (from k = 45 to k = 74), including the top 25% to the top 5% highest-degree nodes of the network. No significant differences between groups were found across this range for neither rich-club, feeder nor local connectivity *strength*. OCD patients displayed significantly reduced rich-club weighted connectivity *density* when rich-club nodes were selected at the top 25% to the top 20% highest-degree nodes (p < .01, Hedge’s g = [0.74,0.85]) (**Figure S2**).

***Threshold .55***. Rich-club organization was found in the white-matter network of both OCD patients and healthy controls (Ø_norm_, range k = 54 to k = 201; Ø_norm_^w^, range k = 82 to k = 194). OCD patients displayed significantly reduced topological rich-club organization (range k = 78 to k = 87, range k = 92 to k = 93, p < .05, uncorrected; Hedge’s g = [0.55,0.69]), as well as significantly reduced weighted rich-club organization (range k = 82 to k = 87, q < .01, Hedge’s g = [0.48,1.04]) (**Figure S1**).

Rich-club nodes selected at the top 16% (k > 118) highest-degree nodes at the group level included the same brain regions reported in the main text. Rich-club, feeder and local connectivity strength and connectivity density were computed across a range of rich-club levels (from k = 103 to k = 151), including the top 25% to the top 5% highest-degree nodes of the network. No significant differences between groups were found across this range for neither rich-club, feeder nor local connectivity *strength*. OCD patients displayed significantly reduced rich-club weighted connectivity *density* when rich-club nodes were selected at the top 25% to the top 10% highest-degree nodes (p < .05, Hedge’s g = [0.60,0.78]) (**Figure S2**).

**Group comparisons including the unaffected siblings**

A final sample of 8 unaffected siblings (mean age 37.8$\pm$13.2 year) was included (see Table 1 for demographic and clinical characteristics). Analysis of rich-club organization was carried out as described in the main text. However, in light of baseline differences in network density and connectivity, only rich-club, feeder and local weighted connectivity *density* were considered (see below). Group comparisons of the observed effects were performed using the Jonckheere-Terpstra test, a nonparametric test for ordered differences in three or more study populations. The permutation version of the test (50,000 permutations) was used for the reference null distribution and the resulting one-sided p-value. Specific hypotheses on the direction of the ordered differences were formulated based on inspection of the data. Analyses were performed in R (<https://CRAN.R-project.org/package=clinfun>).

***Network characteristics.*** The white-matter network of the unaffected siblings presented with significantly lower overall network connectivity and significantly lower network density compared to OCD patients and healthy controls (p < .001, **Table S1**). These group differences were retained for overall network connectivity following the two-step thresholding procedure. However, the direction of the differences reversed for the density of thresholded networks, with the unaffected siblings displaying significantly higher network density compared to OCD and healthy controls (p < .001, **Table S1)**. To avoid introducing any bias, only graph measures including some form of normalization (either by a null distribution or by the overall network connectivity) were considered for the comparisons including the siblings group. Following the two-step thresholding procedure, the networks of all subjects showed to preserve connectedness (> 80% of nodes connected to at least one other node) and small-world topology (small-world index > 1). The unaffected siblings displayed significantly higher small-world properties, but significantly lower global efficiency and global clustering coefficient compared to OCD patients and healthy controls (p < .001, **Table S1**). Differences in local efficiency and local clustering between the unaffected siblings and OCD patients and healthy controls spanned almost the entire network (n = 229 and n = 217 nodes respectively, q < .05).

***Rich-club organization.*** For all three groups of subjects, significant rich-club organization was observed over a range of rich-club levels (Ø_norm_, range k = 20 to k = 53; Ø_norm_^w^, range k = 42 to k = 67). The Jonckheere-Terpstra test revealed significant ordered differences between groups, with HC > OCD > SIB topological rich-club organization (range k = 20 to k = 53, JT = [120, 450], q < .05) and SIB > HC > OCD weighted rich-club organization (range k = 42 to k = 58, JT = [310, 588], q < .05) (**Figure S3**).

***Rich-club, feeder and local connectivity.*** Rich-club nodes selected at the top 16% highest-degree nodes across all groups of subjects (k > 35, n = 64) included the same areas reported in the main text. We found significant ordered differences between groups for rich-club, feeder and local connectivity density. However, the direction of the ordered differences was different across connection classes, with SIB > HC > OCD rich-club connectivity density (JT = 329, p < .001 ); HC > OCD > SIB feeder connectivity density (JT = 384, p < .001) and SIB > OCD > HC local connectivity density (JT = 419, p = .01) (**Figure S3).** This pattern of results was stable across larger and smaller sets of rich-club nodes (range from k > 29 to k > 51, including from the top 25% to the top 5% highest-degree nodes).

**Tables**

**Table S1.** Basic network characteristics and global graph measures of OCD patients, their unaffected siblings and healthy controls.

|  | **OCD patients (n = 28)** | **Siblings (n = 8)** | **HC (n = 28)** |
| --- | --- | --- | --- |
|  | ***Mean (± SD)*** | ***Mean (± SD)*** | ***Mean (± SD)*** |
| *Pre-thresholding* |  |  |  |
| Network connectivity^1^ | 4.5e+09 (±6.7e+08) | 3.2e+09 (±3e+08) | 4.4e+09 (±6.6e+08) |
|  | *diff = 1.3e+09, p < .001* | |  |
|  |  | *diff = -1.2e+09, p < .001* | |
|  | *diff = 1.1e+08, p = 0.53* | |  |
| Network density^2^ | 0.99 (±0.01) | 0.68 (±0.05) | 0.98 (±0.01) |
|  | *diff = 0.31, p < .001* | |  |
|  |  | *diff = -0.31, p < .001* | |
|  | *diff = 0.004, p = 0.26* | | |
| *Post-thresholding* |  |  |  |
| Network connectivity^1^ | 4.3e+09 (±6e+08) | 3.2e+09 (±3e+08) | 4.2e+09 (±5.9e+08) |
|  | *diff = 1.1e+09, p < .001* | |  |
|  |  | *diff = 9.9e+08, p < .001* | |
|  | *diff = 8.3e+07, p = 0.60* | | |
| Network density^2^ | 0.15 (±0.005) | 0.18 (±0.004) | 0.15 (±0.006) |
|  | *diff = -0.03, p < .001* | |  |
|  |  | *diff = 0.03, p < .001* | |
|  | *diff = -1.9e-04, p = 0.91* | | |
| Network connectdeness^3^ | 100% | 100% | 100% |
| Small-worldness | 3.65 (±0.54) | 10.22 (±3.86) | 3.59 (±0.98) |
|  | *diff = -6.58, p < .001* | |  |
|  |  | *diff = 6.64, p < .001* | |
|  | *diff = 0.05, p = 0.82* | | |
| Global efficiency | 4.7e+05 (±6e+04) | 3.9e+05 (±3e+04) | 4.6e+05 (±5e+04) |
|  | *diff = 8.4e+04, p < .001* | |  |
|  |  | *diff = -7.7e+04, p < .001* | |
|  | *diff = 7.5e+03, p = 0.64,* | | |
| Global clustering | 0.012 (±0.002) | 0.006 (±5e-04) | 0.011 (±0.002) |
| coefficient | *diff = 0.006, p < .001* | |  |
|  |  | *diff = -0.005, p < .001* | |
|  | *diff = 0.001, p = .03* | | |
| ^1^ calculated as the sum of edge weights across all nodes; ^2^ calculated as the fraction of present connections to all possible connections, ^3^ percentage of nodes connected to at least one other node. Group comparisons are performed using permutation testing (50,000 permutations of group assignments). The observed difference is calculated in turn as OCD-SIB, SIB-HC, OCD-HC and is assigned a p-value. diff: observed difference; SD: standard deviation; OCD: obsessive-compulsive disorder patients; SIB: unaffected siblings; HC: healthy controls. | | | |

**Table S2.** Local efficiency and local clustering coefficient differences between OCD patients and healthy controls.

|  | **Local efficiency** | **Local clustering coefficient** |
| --- | --- | --- |
| **ID Brainnetome atlas^1^** | ***p-value FDR-corrected (comparison)*** | |
| *Non-rich-club nodes* |  |  |
| 2 - R Superior frontal gyrus (A8m) |  | ** OCD > HC |
| 3 - L Superior frontal gyrus (A8dl) |  | ** OCD > HC |
| 5 - L Superior frontal gyrus (A9l) |  | * OCD > HC |
| 6 - R Superior frontal gyrus (A9l) |  | * OCD > HC |
| 7 - R Superior frontal gyrus (A6dl) |  | * OCD > HC |
| 12 - R Superior frontal gyrus (A9m) |  | * OCD > HC |
| 19 - L Middle frontal gyrus (A46) |  | * OCD > HC |
| 25 - L Middle frontal gyrus (A6vl) |  | * OCD > HC |
| 31 - L Inferior frontal gyrus (IFS) |  | * OCD > HC |
| 34 - R Inferior frontal gyrus (A45c) |  | * OCD > HC |
| 36 - R Inferior frontal gyrus (A45r) |  | * OCD > HC |
| 40 - R Inferior frontal gyrus (A44v) |  | * OCD > HC |
| 43 - L Orbital gyrus (A12/47o) | ** OCD > HC | *** OCD > HC |
| 44 - R Orbital gyrus (A12/47o) |  | ** OCD > HC |
| 45 - L Orbital gyrus (A11l) |  | * OCD > HC |
| 47 - L Orbital gyrus (A11m) | ** OCD > HC | *** OCD > HC |
| 48 - R Orbital gyrus (A11m) |  | * OCD > HC |
| 49 - L Orbital gyrus (A13) |  | * OCD > HC |
| 51 - L Orbital gyrus (A12/47l) |  | ** OCD > HC |
| 52 - R Orbital gyrus (A12/47l) |  | ** OCD > HC |
| 53 - L Precentral gyrus (A4hf) |  | * OCD > HC |
| 58 - R Precentral gyrus (A4ul) |  | * OCD > HC |
| 61 - L Precentral gyrus (A4tl) |  | ** OCD > HC |
| 62 - R Precentral gyrus (A4tl) |  | * OCD > HC |
| 64 - R Precentral gyrus (A6cvl) |  | * OCD > HC |
| 72 - R Superior temporal gyrus (A41/42) | * OCD > HC | *** OCD > HC |
| 79 - L Superior temporal gyrus (A22r) |  | * OCD > HC |
| 82 - R Middle temporal gyrus (A21c) | * HC > OCD | * HC > OCD |
| 91 - L Inferior temporal gyrus (A37elv) |  | * OCD > HC |
| 92 - R Inferior temporal gyrus (A37elv) |  | * OCD > HC |
| 95 - L Inferior temporal gyrus (A20il) |  | * OCD > HC |
| 96 - R Inferior temporal gyrus (A20il) | ** HC > OCD | *** HC > OCD |
| 100 - R Inferior temporal gyrus (A20cl) |  | ** OCD > HC |
| 101 - L Inferior temporal gyrus (A20cv) |  | * OCD > HC |
| 102 - R Inferior temporal gyrus (A20cv) |  | *** OCD > HC |
| 112 - R Parahippocampal gyrus (A35/36c) |  | * OCD > HC |
| 116 - R Parahippocampal gyrus (A28/34) |  | * OCD > HC |
| 120 - R Parahippocampal gyrus (TH) |  | * OCD > HC |
| 124 - R Posterior superior temporal sulcus (cpSTS) | ** HC > OCD | * HC > OCD |
| 125 - L Superior parietal lobule (A7r) |  | * OCD > HC |
| 130 - R Superior parietal lobule (A5l) | ** OCD > HC | *** OCD > HC |
| 134 - R Superior parietal lobule (A7ip) | ** OCD > HC | *** OCD > HC |
| 137 - L Inferior parietal lobule (A39rd) |  | * OCD > HC |
| 157 - L Postcentral gyrus (A1/2/3tonla) |  | * OCD > HC |
| 159 - L Postcentral gyrus (A2) | ** OCD > HC | *** OCD > HC |
| 171 - L Insular gyrus (dlg) |  | * OCD > HC |
| 183 - L Cingulate gyrus (A24cd) |  | * OCD > HC |
| 184 - R Cingulate gyrus (A24cd) |  | * OCD > HC |
| 186 - R Cingulate gyrus (A23c) |  | * OCD > HC |
| 188 - R Cingulate gyrus (A32sg) |  | ** OCD > HC |
| 193 - L Medioventral occipital cortex (cCunG) |  | ** OCD > HC |
| 199 - L Lateral occipital cortex (mOccG) |  | ** OCD > HC |
| 200 - R Lateral occipital cortex (mOccG) | * OCD > HC | *** OCD > HC |
| 205 - L Lateral occipital cortex (iOccG) |  | * OCD > HC |
| 212 - R Amygdala (mAmyg) |  | * OCD > HC |
| 229 - L Dorsolateral putamen |  | * OCD > HC |
| 233 - L Thalamus (mPMtha) |  | ** OCD > HC |
| 240 - R Thalamus (PPtha) |  | * OCD > HC |
| 245 - L Thalamus (lPFtha) |  | * OCD > HC |
| 247 - L Subthalamic nucleus |  | ** OCD > HC |
|  |  |  |
| *Rich-club nodes^2^* |  |  |
| 10 - R Superior frontal gyrus (A6m) |  | ** OCD > HC |
| 27 - L Middle frontal gyrus (A10l) |  | * OCD > HC |
| 37 - L Inferior frontal gyrus (A44op) |  | * OCD > HC |
| 73 - L Superior temporal gyrus (TE1.0/1.2) |  | * OCD > HC |
| 77 - L Superior temporal gyrus (A38l) |  | * OCD > HC |
| 107 - L Fusiform gyrus (A37lv) |  | *** OCD > HC |
| 135 - L Inferior parietal lobule (A39c) |  | ** OCD > HC |
| 136 - R Inferior parietal lobule (A39c) |  | * OCD > HC |
| 138 - R Inferior parietal lobule (A39rd) |  | * OCD > HC |
| 150 - R Precuneus (A5m) |  | *** OCD > HC |
| 181 - L Cingulate gyrus (A23v) |  | * OCD > HC |
| 197 - L Medioventral occipital cortex (vmPOS) |  | * OCD > HC |
| 217 - L Hippocampus (cHipp) |  | * OCD > HC |
| 220 - R Ventral caudate |  | * OCD > HC |
| *^1^* ID, hemisphere, gyrus and cytoarchitectonic description reported as in the Brainnetome Atlas. *^2^* Rich-club nodes across all sets considered (top 25% to top 5%). Group comparisons are performed using permutation testing (50,000 permutations of group assignments). The observed difference is calculated as OCD-HC and is assigned a p-value. All reported p-values are FDR-corrected at q = 0.05. *** q < 0.001; ** q < 0.01; * q < 0.05; L: left; R: right; OCD: obsessive-compulsive disorder patients; HC: healthy controls. | | |

**Table S3.** Partial correlation coefficients between observed effects and clinical characteristics.

**Figures**

**Figure S1.** The group average normalized Ø (*A, B top*) and Ø^w^ (*A, B bottom*) are plotted for OCD patients (purple) and healthy controls (yellow) for the proportional thresholding parameters of .30 (*A*) and .55 (*B*). Normalized Ø > 1 (dashed line) indicates significant rich-club organization. The grey shaded area indicates where rich-club organization of the two groups is significantly different (q < 0.05). The lighter grey shaded area in panel B (*top*) indicates uncorrected p-values (p < 0.05). OCD: obsessive-compulsive disorder patients; HC: healthy controls; th: proportional threshold.

**Figure S2.** The difference between the rich-club, feeder and local connectivity strength (*A*) and density (*B*) of OCD patients and healthy controls is plotted for the networks constructed using a proportional threshold of .23 (*A,B top*), .30 (*A,B middle*) and .55 (*A,B bottom*). The connectivity measures are calculated over a range of rich-club levels *k* that always include from the top 5% to the top 25% highest-degree nodes of the network. Between-group differences are observed across proportional thresholding parameters for a range of k levels for rich-club connectivity density (blue asterisks in B).

*: p < 0.05; OCD: obsessive-compulsive disorder patients; HC: healthy controls; th: proportional threshold.

**Figure S3.** Individual normalized Ø and Ø^w^ are plotted for OCD patients (purple), healthy controls (yellow) and unaffected siblings (green) for different rich-club levels. Normalized Ø > 1 (dashed line in *A* and *B*) indicates significant rich-club organization. The grey shaded area indicates significant ordered differences between groups (HC > OCD > SIB, q < 0.01, *A*) (SIB > HC > OCD, q < 0.05, *B*). Rich-club nodes are selected at the top 16% highest-degree nodes of the network (k > 35), and network edges are classified accordingly into rich-club, feeder and local (not shown). Rich-club, feeder and local connectivity density are tested for ordered differences between groups (*C*). ***: p < 0.001; **: p < 0.01; OCD: obsessive-compulsive disorder patients; HC: healthy controls; SIB: unaffected siblings.
